# Global Research Trends on the Treatment of Diffuse Large B-Cell Lymphoma: A Bibliometric and Visualized Study

**DOI:** 10.7150/jca.68453

**Published:** 2022-03-14

**Authors:** Qintong Duan, Yufeng Li, Lijia Ou, Yajun Li, Ruolan Zeng, Yizi He, Tao Pan, Siwei Chen, Huan Chen, Hui Zang, Hui Zhou, Ling Xiao

**Affiliations:** 1 Department of Histology and Embryology of School of Basic Medical Sciences, Central South University, Changsha, Hunan, China.; 2 Department of Lymphoma & Hematology, The Affiliated Tumor Hospital of Xiangya Medical School, Central South University, Changsha, Hunan, China.; 3 Department of Pathology and Pathophysiology of School of Basic Medical Sciences, Hunan University of Medicine, HuaiHua 418000.; 4 Department of Human Anatomy and Histoembryology of School of Basic Medical Sciences, Yiyang Medical College, Yiyang.

**Keywords:** diffuse large B-cell Lymphoma (DLBCL), treatment, research progress, bibliometric analysis, Visualized, VOSviewer

## Abstract

Diffuse large B-cell lymphoma (DLBCL) is the most common lymphoma subtype. Treatment of DLBCL has improved greatly in recent decades, with thousands of papers published. We conducted a bibliometric analysis of the literature on DLBCL treatment, and discussed cooperation among authors, countries, and institutions, and identified research hotspots for DLBCL treatment. We searched the Web of Science Core Collection (WOSCC) using “Diffuse Large B Cell Lymphoma or DLBCL” and “Treatment or Therapy or Clinical Trial” as the subject terms, and analyzed the publication year, research direction, country/region, institution, author, source publication, distribution of funding institutions, and other conditions provided by the database. In addition, scientometrics software was used to analyze literature citations and cooperative publications. Bibliometric analyses were performed using https://bibliometric.com/app and VOSviewer. Network maps were generated to evaluate collaborations between different authors, countries, institutions, and keywords. A total of 7,255 studies on treatment of DLBCL were retrieved from the WOSCC on February 19, 2021. We found that the number of publications increased gradually from 1999 to 2021, and this trend was relatively stable in the past 3 years. The countries that produced the most publications were the United States, China, and Japan. Among institutions, University of Texas MD Anderson Cancer Center published the most manuscripts. Furthermore, the United States also had the most annual publications, citations, distribution of journal sources, and funding. Cooperative research between countries is also relatively important to treatment of DLBCL. Therapeutic regimens such as CHOP and R-CHOP, and immunotherapy (CAR-T, PD1/PDL1, and CAR-NK, etc.), have received increased attention. Bibliometric analysis of studies related to DLBCL treatment can help researchers and clinical workers quickly understand the hotspots and development trends in this field, and provide reference for the formulation of public health policies.

## Introduction

Diffuse large B-cell lymphoma (DLBCL) is the most common lymphoma subtype, and accounts for 30%-40% of adult non-Hodgkin's lymphoma cases 1. Survival rates have improved over the past few decades, with recent relative survival rates reported as 55.4% in Europe and 62.0% in the Americas 2. In the era of immunochemotherapy, rituximab combined with cyclophosphamide, doxorubicin, vincristine, and prednisone (R-CHOP) cured more than 50% of patients with advanced-stage de novo DLBCL 3. Only 30% to 40% of patients who progress during primary immunochemotherapy or after a brief period of CR will respond to salvage chemotherapy and may subsequently receive autologous stem cell transplantation (ASCT) consolidation therapy 4-6. Furthermore, among patients with relapsed or refractory diffuse large B-cell lymphoma who respond to salvage therapy and are able to undergo ASCT, approximately 50% of patients eventually relapse after transplantation 7, 8. These patients have a poor prognosis, especially those with high risk factors such as relapse 12 months after ASCT or secondary IPI score of 2 6, 9. There are no effective treatment options for the majority of patients with refractory DLBCL 10.

Development of methods such as cytogenetics, cellular immunology, and molecular biology has allowed for screening of different DLBCL subtypes and development of targeted immunotherapy based on the more accurate stratification method, which has brought new hope to DLBCL patients, particularly those with relapsed/refractory DLBCL (R/R DLBCL). To accurately understand the global research status of DLBCL therapy, methods such as bibliometrics are needed to synthesize and analyze research progress, which will allow for better access reference information for researchers.

Bibliometric analysis is a method that can quantify research interests and hot topics in the research community 11. Bibliometric analysis refers to the use of mathematical and statistical methods to quantitatively analyze all the knowledge carriers of a certain discipline. This information is used to describe, evaluate, and predict current and future trends in science and technology 12. Bibliometrics is widely used in the field of medical research because it can provide reference data for understanding research and technological development, determining scientific topics for research, evaluating the novelty of projects, and promoting research results 12, 13. Bibliometric studies have been conducted to examine trends in medical research output for gastroenterology 14, infectious diseases 15, 16, microbiology 17, oncology 18, 19, otolaryngology 20, respiratory medicine 21, surgery 22, and public health 23-25.

We conducted a quantitative study of the science citation index (SCI) articles included in the WoSCC of IS Web of Knowledge, analyzed the global publication landscape, and identified influential journals and studies in this field. The purpose is to facilitate DLBCL research through identification of research topics, published research results, and research partners.

## Materials and Methods

### Data Sources

Literature data were obtained from the Science Citation Index Expanded (SCIE), the most influential database for obtaining scientific and technological academic information in the world. Through SCIE, researchers can access the latest research papers from more than 8,000 academic journals from thousands of publishing houses around the world, and access research ideas based on existing research results through a 100-year retrospective database. In addition, SCIE also links papers through cross-referencing between papers. Through use of hyperlinks, researchers can easily trace references cited in manuscripts and papers that cite their publications, which can help to identify previous findings and influence subsequent research directions.

### Search Methods and Bibliometric Analysis

We conducted a literature search using the Web of Science Core Collection (WoSCC), limited to Science Citation Index-Expanded (SCIE), to identify DLBCL-related publications from the past decades with no language restriction. The search included all document types including original articles, reviews, letters, and editorials. The following documents were excluded: (1) recovered publications, (2) retracted articles, and (3) duplicate papers. Our search terms were “diffuse large B cell lymphoma or DLBCL” AND “treatment or therapy”. A total of 7,255 related publications were retrieved from 1999 to date. All retrieved records were downloaded on February 19, 2021 and imported into bibliometric tools for further analysis. All records and cited references were stored in “.txt” format. The documents saved in “.txt” format were opened using the online analysis platform “https://bibliometric.com/”, and analyzed for countries and regions, institutions, authors, source publications, fund-supported institutions, research directions, and annual published documents. The Visualize Network and Density functions in VOSviewer software were used to objectively understand the research hotspots in this field. Finally, VOSviewer software was used to analyze the co-occurrence of author's individual cooperation network, author's organization cooperation network and author's country-region cooperation network 26, 27, to allow for more extensive scientific research. In basic retrieval mode, different types of therapeutic methods and DLBCL were used as keywords for the scientific citation index.

## Results

### Publication of international articles

**Figure 1:**
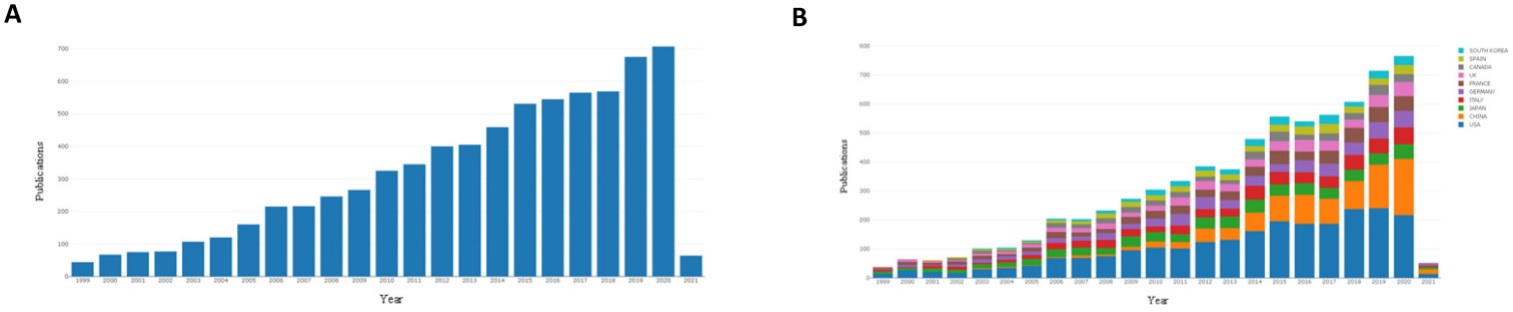
Published articles in the field of DLBCL therapy in recent years. **(A):** Annual articles published in the research field of diffuse large B lymphoma in the past 20 years. **(B):** The status of articles published in the top 10 countries in the field of treatment of diffuse large B lymphoma in recent years.

We began with an analysis of the international literature published in the last two decades. Articles published worldwide from 1999-2021 covering the research field of treatment of DLBCL were searched (Figure 1A). As shown in Figure 1a, the number of publications increased year over year. Forty-four articles were published in 1999, and 707 articles were published in 2020. This indicated that global research in the field of treatment of DLBCL has received increasing attention.

The research fields for DLBCL treatment were ranked by countries, and the top ten countries with published studies were the United States, China, Japan, Italy, Germany, France, Britain, Canada, Spain, and South Korea. Since 1999, the number of publications in many countries has increased (Figure 1B). A total of 2,389 papers (33.26%) were published in the United States, which was the most, and 996 papers (13.45%) and 643 papers (8.96%) were published in China and Japan, respectively (Table 1). This result demonstrated that the United States has contributed most strongly to the literature related to treatment of DLBCL, and highlights the attention paid to this emerging field. These results showed that the United States, Japan, China, and Europe were the main countries and regions that contributed to the literature on treatment of DLBCL.

### Ranking of institutions, distribution of source journals, and citations of articles

Scientific research is conducted by scientific institutions. Among the 10 institutions with the most published articles in the field of treatment of DLBCL, nine are from the United States and one is from Canada. This indicated that the United States has placed extensive focus on research into treatment of DLBCL. The University of Texas MD Anderson Cancer Center in the United States ranked first with 409 publications. Memorial Sloan Kettering Cancer Center and Mayo Clinic ranked second and third in terms of publications, respectively (Table 2).

The source publications were ranked according to the number of articles included. The top 3 journals related to lymphoma, “Blood”, “Leukemia & Lymphoma”, and “British Journal of Haematology” have published 528 (7.35%), 409 (5.695%), and 214 (2.98%) articles on treatment of DLBCL, respectively, and ranked in the top three. The “Journal of Clinical Oncology”, was fourth, and published 206 articles (2.88%), and “ANNALS OF ONCOLOGY” was fifth with 178 articles (2.48%). The top 10 were all oncology- and hematology-related journals, and the journal impact factors ranged from 2.22-32.956, which indicated that these manuscripts were published in influential journals (Table 3).

The most cited article “Distinct types of diffuse large B-cell lymphoma identified by gene expression profiling” (6,598 total citations) was published in Nature in 2000, and the second and third-ranked articles were published in The New England Journal of Medicine (Table 4). The first authors and corresponding authors of these articles were all European and American scientists, which highlights the focus of researchers in these regions on this topic.

### Distribution of Funding Institutions

**Figure 2:**
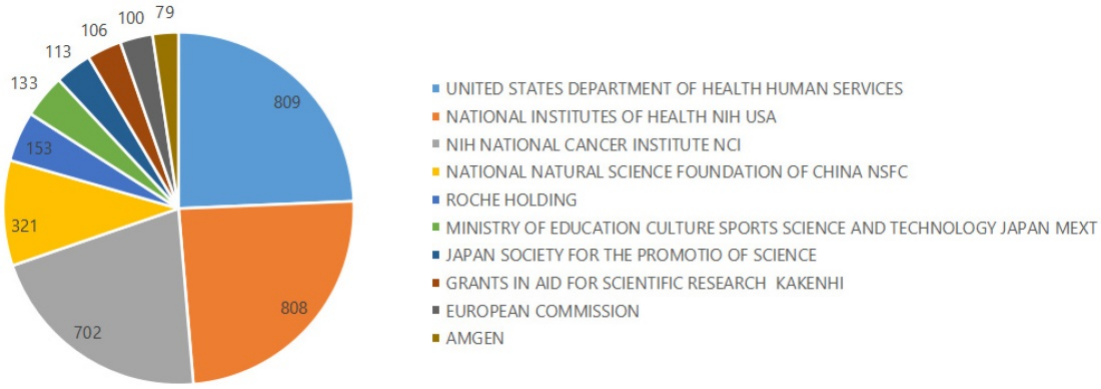
Top 10 funding agencies and the number of documents issued.

The papers that result from funded projects often reflect and importance of certain research field, and also represent the development trends, scientific research resources, and scientific research level of the research field 28. Ten funding agencies, including the United States Department of Health and Human Services, have contributed significantly to DLBCL therapeutic research (Figure 2). Although there is no identifiable correlation between the level of funding and the level of knowledge output, the United States has disproportionately led research in the field focused on treatment of DLBCL, as the top three funding institutions were all from the United States.

### Literature citation

**Figure 3:**
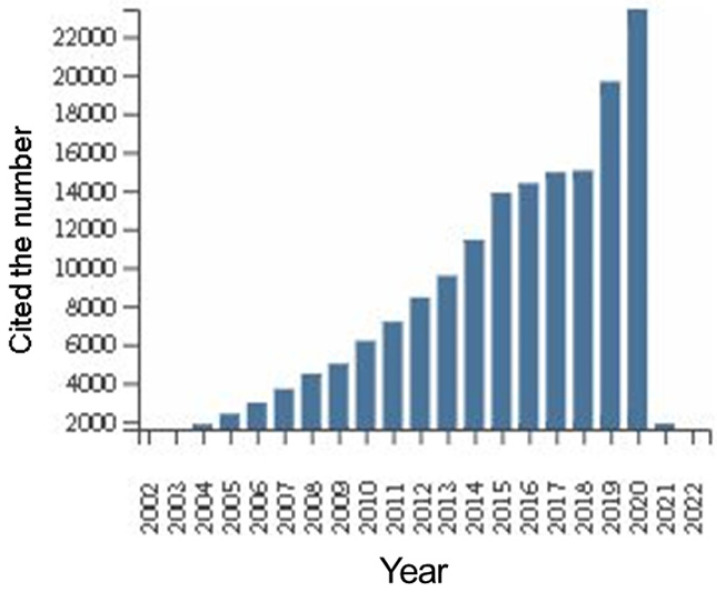
Global citations of DLBCL literature from 1999 to 2021.

We performed additional analysis of the citation status of all documents in this field of research worldwide (Figure 3). The citation frequency of manuscripts has increased year over year for the past 20 years, with slow growth from 2014 to 2018, but a sudden increase in the number of papers cited in 2019 and 2020, which indicated increased focus on this field. The citations of documents in this are shown in Table 5. The average number of citations and h-index for each item (defined as: sorting papers from high to low by citation frequency, h-index is the number of citing documents for papers ranked as “h” is greater than or equal to “h”) are the two most important indicators. The average number of citations for each item reflects the influence of an average single article. The average number of citations for each item was 23.33. The h-index is the comprehensive result of the number of articles and the number of citations, which can reflect the number and quality of articles. The h-index was 154.

### Visual analysis of articles related to DLBCL treatment

**Figure 4:**
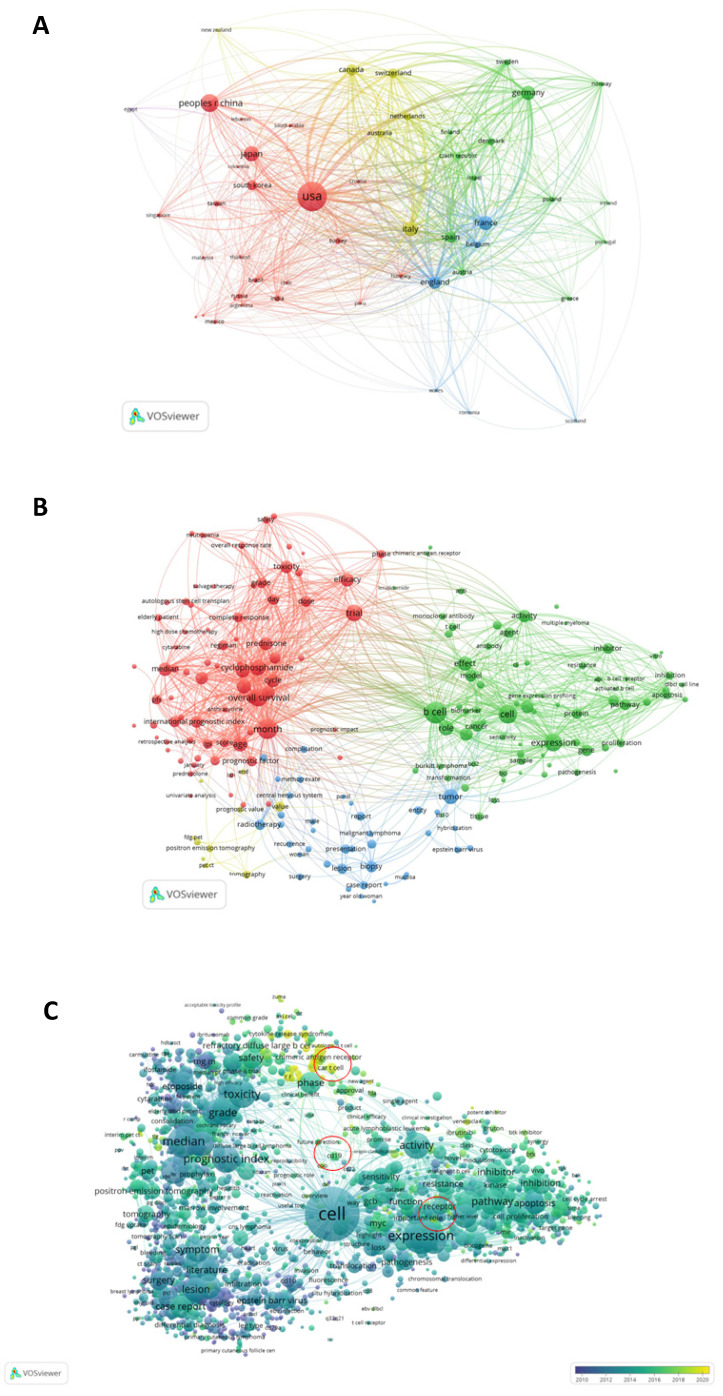
** VOSviewer was used to analyze the obtained literature. (A):** Overlay visualization map of country co-authorship analysis. Countries were represented by circle labels. The distance between two circles indicated the closeness of relationships. The strength of the co-author link between two countries was represented by the thickness of the connecting curved lines. The area of the circle was determined by the number of total citations by each country. **(B):** Overlay visualization map of author keywords co-occurrence analysis. The area of the circle was determined by occurrences of each keyword, and the same color represents the same cluster, with brighter colors indicated more research focused on these keywords. **(C):** In the visual analysis of keyword overlay, time was added to the keyword co-occurrence network, which clearly shows the research focus and direction of this field in different time periods. Different colors of circles indicates the average year of the studies according to the bar on the lower right corner.

Figure 4A shows the analysis results of the country-region cooperation network of authors who have co-published 25 or more articles. The size of the circle in the figure shows the degree of cooperation between countries, and the width of the line connecting the countries represents the strength of cooperation. The United States, Japan, China, Canada, Germany, France, and many other countries have strong cooperative relationships.

Keyword co-occurrence cluster analysis was performed using VOSviewer software, and current research hotspots in this field were determined. The results were as follows: (1) Prognostic factors (red): toxicity, efficacy, overall survival, retrospective analysis, score, age, etc.; (2) Mechanism research (green): expression, model, biomarker, pathway, gene, etc.; (3) Clinical indicators (blue): biopsy, radiotherapy, lesion, recurrent, Surgery, etc.; (4) Pathological indicators (yellow): value, PET CT, tomography, etc. (Figure 4B). Overlay visual analysis of keywords was performed to evaluate time and keyword co-occurrence (Figure 4C). In recent years, immunotherapy has emerged as a treatment for DLBCL. For example, CAR-T therapy has received increasing attention in recent years, as shown in the circles in Figure 4C. In addition, Targeting CD19, a form of CAR-T cell therapy has shown great promise.

### Analysis of the progress of typical immunotherapies for DLBCL

We divided the progress of DLBCL treatment into chemotherapy drugs, small molecule compounds, monoclonal antibodies (mAb), cell therapy, and transplantation, and ranked each field based on the number of national papers. The statistical results are shown in Table 6. The United States was the leader in DLBCL therapy research, and China and Italy were among the leaders in research focus on chemotherapy drugs. China and Spain were the leaders in evaluation of small molecule compounds. Monoclonal antibody research was a major focus in France and Britain, and the UK and Italy were leaders in research focused on transplant.

**Figure 5:**
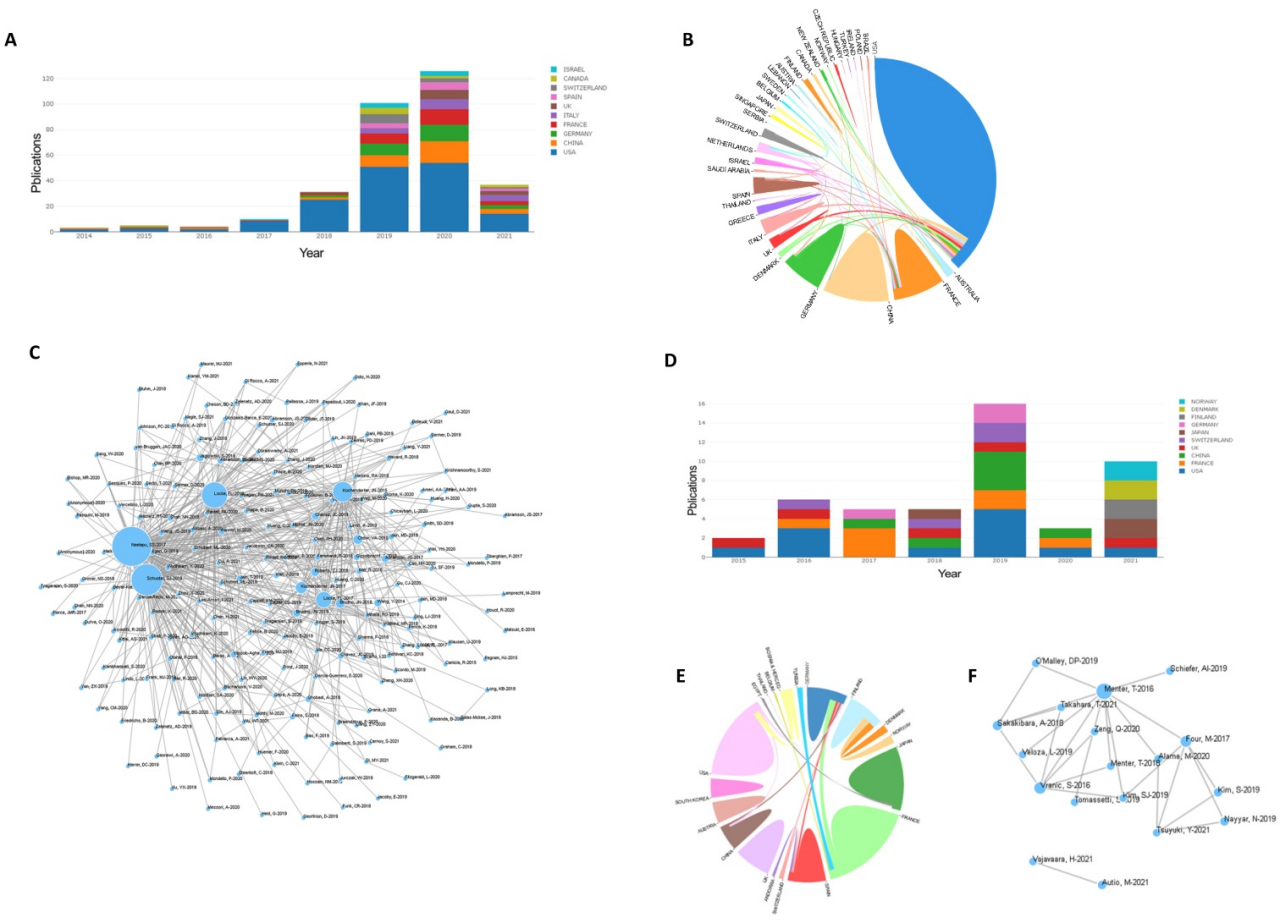
** (A,B,C):** Statistics on CAR-T therapy. (D,E,F): Statistics on PD1/PDL1 therapy. **(A,D):** Annual articles published on use of CAR-T and PD1/PDL1 for DLBCL in various countries. **(B,E):** Cooperative relations between countries. Countries are represented in different colors. The strength of the cooperation between co-authors between two countries was expressed by the thickness of the connection curve. The percentage area was determined by the total number of citations per country. **(C,F):** Relational networks. Larger dots indicate higher quality manuscripts and number of citations.

Next, we focused on the research status of CAR-T and PD1/PDL1 for treatment of DLBCL. A total of 281 articles focused on CAR-T in DLBCL were retrieved. Statistical analysis found that relevant articles began to appear in 2014, and the number of articles published has steadily increased since then (Figure 5A). There were strong links between many countries but there were few academic exchanges between countries with abundant publications and countries with weak publications (Figure 5B). The reference network of the articles is shown in Figure 5C and focuses on three highly cited articles of high quality, “Locke FL, et al. Long-term safety and activity of axicabtagene ciloleucel in refractory large B-cell lymphoma (ZUMA-1): a single-arm, multicenter, phase 1-2 trial. Lancet Oncol. 2019 Jan; 20(1): 31-42”, “Neelapu SS, et al. Axicabtagene Ciloleucel CAR T-Cell Therapy in Refractory Large B-Cell Lymphoma”, and “Schuster SJ, et al. Tisagenlecleucel in Adult Relapsed or Refractory Diffuse Large B-Cell Lymphoma. N Engl J Med. 2019 Jan 3”, which indicates that these manuscripts are among the most influential in the field. A total of 42 articles focused on PD1/PDL1 in DLBCL were retrieved. Statistical analysis found that relevant articles appeared in 2015 and research has continued to focus on PD1/PDL1 treatment (Figure 5D). Our results showed strong links between countries (Figure 5E). Few studies were found that focused on PD1/PDL1 mAb treatment for DLBCL, which may be related to poor efficacy of this treatment. A key article, “Menter T, et al. Evaluation of the diagnostic and prognostic value of PDL1 expression in Hodgkin and B-cell lymphomas. Hum Pathol. 2016 Aug; 54: 17-24” has been cited extensively.

## Discussion

Bibliometric analysis has developed into the best tool for exploring trends in detailed research in a specific area 29. It objectively presents the research contributions of different countries, institutions, and journals in related fields through qualitative and quantitative analysis, and predicts research hotspots, and trends, resulting in identification of unsolved problems in specific fields, or important research directions that should be evaluated. In addition, bibliometric analysis plays an important role in future development of policy and clinical guidelines for various diseases 30. However, no bibliometric analysis of DLBCL and treatment strategies had been performed previously, and little attention had been paid to prediction of research hotspots. This was a quantitative and qualitative bibliometric study of treatment of DLBCL, and used literature on treatment of DLBCL from the past two decades.

The United States was the leading contributor in the DLBCL treatment field with 2,389 publications, followed by China, Japan, Italy, Germany, France, Britain, Canada, Spain, and South Korea. Seven of the top 10 are developed countries are from developed countries in Europe and the Americas, while only China, Japan, and South Korea were from Asia. Moreover, the published literature from Asia was relatively less cited, despite the population of Asia accounting for about 60% of the world population 31. The number of papers in this field has been rapidly growing since 1999 and has remained relatively stable in the past 3 years. This finding indicated that literature output in this field should continue at a similar rate over the next few years. Papers published in specialty journals attract particular attention, and most papers on DLBCL treatment are published in international core journals. The number of publications in the United States, China, Italy, and other countries showed an increasing trend, which may be related to open and reasonable academic policies, national conditions, and economic growth, resulting in better financial support for research 32, 33. In addition, funding mainly originated from national funds, and the funding history indicated that DLBCL research has received extensive attention. To narrow the gap between countries in this field of research, the following strategies may promote increased focus on research in countries with less strong research records: a. Strengthen the popularization of scientific knowledge and academic research, and strengthen societal respect for knowledge and technology; b. Improve the evaluation system for researchers, innovate research technologies and methods, and improve the quality of published papers. In addition, place focus on the number of articles published, and increase the influence of authoritative journals; c. Government agencies should increase investment in scientific research, and increase investment in training of strong researchers 34; d. Strengthen international collaboration among countries, institutions, and authors to increase the number of high-quality publications.

Diffuse large B cell lymphoma (DLBCL) represents the most frequent type of B cell non-Hodgkin lymphoma (B-NHL) in the adult population worldwide. It is characterized by a high degree of genetic complexity and molecular heterogeneity 35, 36. This heterogeneity is a barrier to a 'one size fits all' approach for novel treatments for DLBCL 37, and highlights the need to develop diverse treatment options and to target multiple molecular pathways. We listed the most cited studies and the latest high-quality manuscripts, and summarized the progress of treatment research and representative clinical trials for DLBCL to highlight the current research status in this field. In 2006, the U.S. Food and Drug Administration (FDA) approved rituximab for use as a first-line treatment for patients with DLBCL in combination with CHOP (cyclophosphamide, doxorubicin, vincristine, and prednisone), which has been used for more than 20 years. This regimen, R-CHOP, has become the new standard of treatment for patients with DLBCL, despite lack of efficacy of R-CHOP against the non-germinal center B-cell (non-GCB) subtype of DLGCL as compared to that against GCB subtype 38. In recent years, the R-CHOP + X model has been adopted, in which randomized clinical trials have added new, targeted drugs to R-CHOP to efficacy. Drugs added to R-CHOP include bortezomib 39 and lenalidomide 40. Several phase III studies have failed 41. Several approaches and agents have received FDA approval for treatment of DLBCL including polatuzumab vedotin, tafasitamab, selinexor, axicabtagene ciloleucel, and tisagenlecleucel. Furthermore, development of immunotherapies has continued to advance. A promising cellular immunotherapy for treatment of relapsed/refractory (r/r) DLBCL is chimeric antigen receptor (CAR) T cells. The potent therapeutic efficacy of axicabtagene ciloleucel (axi-cel, marketed as Yescarta) 42, lisocabtagene maraleucel 43, and tisagenlecleucel (marketed as Kymriah) 44 has been demonstrated of CD19-directed CAR T cell therapy. Similarly, genetically modified allogeneic NK cells are another promising option for CAR T cell therapy. The safety of allogeneic NK cells after infusion for adaptive immunotherapy in patients with cancer has been demonstrated 45, 46. Furthermore, CAR NK cells have been shown to induce a response in patients with high-risk CD19-positive cancers with relatively few adverse events 47. Immunomodulatory drugs and immune checkpoint inhibitors (ICIs) have also used to treat DLBCL. For example, lenalidomide has been shown to be effective as a monotherapy for treatment of r/r DLBCL 48, or in combination with salvage chemotherapies, such as R-ICE 49 and R-ESHAP 50. Immune checkpoint inhibitors include pembrolizumab and nivolumab for PD-1, and durvalumab, avelumab, and atezolizumab for PD-L1 51. As medical research receives more attention, novel treatment methods continue to emerge. Molecular pathway inhibitors have been developed to inhibit the BCR signaling pathway inhibition, BCL‑2, VEGFR, PI3K/Akt/mTOR, NF‐κB, JAK/STAT3, and nuclear export pathways. Epigenetic-modifying drugs such as histone deacetylase inhibitors, enhancer of zeste homolog 2 (EZH2) inhibitors, and bromodomain inhibitors have been developed 52.

**Figure 6:**
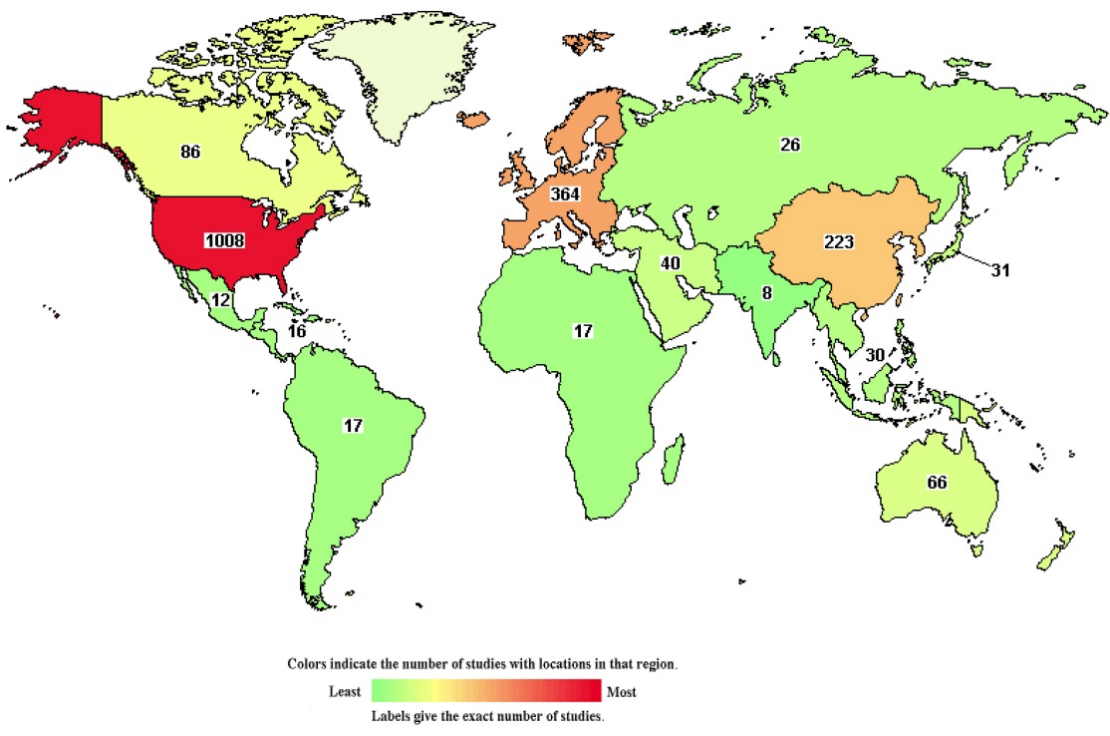
The chart shows 1,537 studies on DLBCL in the clinical trials database. The colors indicate the number of studies located in each region (green to red indicates the number of studies from low to high).

Clinical trials of many new drugs are in progress. A total of 1,537 studies on DLBCL were found in the Clinical Trials Database, https://clinicaltrials.gov/, and were accessed 26 May 2021 (Figure 6). According to the Clinical Trials Database, zanubrutinib, a BTK inhibitor, has been used in research projects at several institutions (ClinicalTrials.gov Identifier: NCT04835870, NCT04460248), and several studies have been linked to clinical trials of lenalidomide (ClinicalTrials.gov Identifier: NCT03715296, NCT04432714). At the 2021 ASCO Annual Meeting in Chicago (United States; June 4-8, 2021), recent research developments regarding DLBCL were reported. A study by Catherine S. Magid Diefenbach et al. evaluated the combination of polatuzumab vedotin (Pola) + rituximab (R) + lenalidomide (Len) (Pola-R-Len), and demonstrated a tolerable safety profile. This first efficacy report of Pola-R-Len showed promising progress toward treatment of R/R DLBCL, particularly with regard to patients achieving complete remission, many of whom remained in remission at the cutoff date. Johannes Dull et al. showed that combination therapy with tafasitamab + lenalidomide (LEN) followed by tafasitamab monotherapy provided positive and lasting responses in patients with R/R DLBCL who were not eligible for autologous hematopoietic stem cell transplantation (ASCT), with a manageable safety profile. These drugs may become the focus of clinical attention in the future. However, there have also been some negative results in phase III randomised trials conducted over the past 2 decades. For example, dose-adjusted EPOCH (poposide, prednisone, vencristine, cyclophosphaamide, and doxorubicin) combined with tuximab (DA-EPOCH-R), failed to show an improvement in survival outcomes in patients with DLBCL (CALGB 50303) 41. In patients with ABC-DLBCL from ROBUST, addition of lenalidomide to R-CHOP did not improve efficacy over placebo/R-CHOP, and the primary end point of progression-free survival (PFS) and the key secondary efficacy end point EFS were not met 53. In a randomized phase III trial of ibrutinib and rituximab plus R-CHOP in non-germinal center B-cell DLBCL, the primary end point of event-free survival (EFS) was not met in the intent-to-treat (ITT) population or the activated B-cell (ABC) DLBCL population. However, in patients younger than 60 years, ibrutinib plus R-CHOP improved EFS, PFS, and overall survival (OS), with tolerable safety. In patients 60 years of age and older, ibrutinib plus R-CHOP was associated with increased toxicity, leading to compromised R-CHOP administration and worse outcomes 54. Negative results may be related to patient baseline characteristics, pharmacokinetics, dose, mutation status, and other potential factors. However, these findings indicate that increased focus has been placed on development of new treatment strategies for DLBCL, an in particular R/R DLBCL.

Our study had several strengths. First, this study used bibliometric analysis for the first time to provide an in-depth understanding of the global research status and trends in DLBCL treatment. Second, we conducted our study with widely used tools to ensure the reliability of the data and to ensure that the analysis was objective and comprehensive. However, similar to other bibliometric analyses, our study suffered from the following limitations. First, we only investigated publications in the WoSCC database. Other databases such as PubMed and Cochrane Library were not searched, which may have resulted in omission of some publications. Second, bibliometric analysis results may differ from the results of research studies. Our results are from published studies, but some important information may be published in forms other than scientific publications. Despite these limitations, this study provides a solid global perspective on research in the DLBC field over the past two decades.

In conclusion, this was the first study to use bibliometrics and visual analysis to identify countries, institutions, authors, and journals that have made significant contributions in the field of DLBCL therapy, and to discuss the general trends and future hot spots in the field. What's more, we summarize the research progress of DLBCL treatment. Multidrug resistance and heterogeneity of DLBCL highlight the need to develop novel alternative strategies such as molecular targeted drugs and immunotherapies to replace traditional chemotherapy. Ongoing clinical trials and a 20-year literature search also confirmed that it is imperative to explore new therapies to overcome the poor prognosis associated with DLBCL heterogeneity. This study provides excellent guidance for DLBCL research, and the hotspots highlighted might contribute to a breakthrough in development of novel treatment strategies and provide ideas for development and improvement of public health policies.

## Figures and Tables

**Table 1:** Ten most productive countries 1999-2021

Standard Competition Ranking	Country	Articles (%)
First	the United States	2389(33.26%)
Second	China	966(13.45%)
Third	Japan	643(8.95%)
Fourth	Italy	582(8.10%)
Fifth	Germany	571(7.95%)
Sixth	France	543(7.56%)
Seventh	England	471(6.56%)
Eighth	Canada	357(4.97%)
Ninth	Spain	342(4.76%)
Tenth	Korea	302(4.20%)

**Table 2:** Top 10 institutions for publications

Institutions	Frequency	Total Cited (times)	Average Cited (times)
Univ Texas MD Anderson Canc Ctr	409	3723	9.10
Mem Sloan Kettering Canc Ctr	350	4113	11.75
Mayo Clin	344	4291	12.47
NCI	238	10361	43.53
Stanford Univ	229	9986	43.61
British Columbia Canc Agcy	150	6279	41.86
Univ Penn	149	1099	7.38
Sungkyunkwan Univ	147	811	5.52
Natl Canc Ctr	144	769	5.34
Fudan Univ	144	367	2.55

**Table 3:** Top 10 journals published in literature

Journal	Frequency	Total Cited (times)	Average Cited (times)	IF (2020)
BLOOD	582	6417	11.03	23.629
LEUKEMIA & LYMPHOMA	406	1773	4.37	3.28
BRITISH JOURNAL OF HAEMATOLOGY	214	1422	6.64	6.998
JOURNAL OF CLINICAL ONCOLOGY	206	6096	29.59	44.544
ANNALS OF ONCOLOGY	178	1997	11.22	32.976
ANNALS OF HEMATOLOGY	166	782	4.71	3.673
HAEMATOLOGICA	150	463	3.09	9.941
CLINICAL LYMPHOMA MYELOMA & LEUKEMIA	114	279	2.45	3.231
INTERNATIONAL JOURNAL OF HEMATOLOGY	96	263	2.74	2.49
EUROPEAN JOURNAL OF HAEMATOLOGY	86	409	4.76	2.997

**Table 4:** Top 10 articles with total citations

Title	Author	Details	Total Cited (times)	Average Cited (times)
Distinct types of diffuse large B-cell lymphoma identified by gene expression profiling	Alizadeh AA, Eisen MB, Davis RE, Ma C, Lossos IS, Rosenwald A, et al.	Nature. 2000;403(6769):503-11	6598	314.19
CHOP chemotherapy plus rituximab compared with CHOP alone in elderly patients with diffuse large-B-cell lymphoma	Coiffier B, Lepage E, Briere J, Herbrecht R, Tilly H, Bouabdallah R, et al.	N Engl J Med. 2002;346(4):235-42	3591	189.00
The use of molecular profiling to predict survival after chemotherapy for diffuse large-B-cell lymphoma	Rosenwald A, Wright G, Chan WC, Connors JM, Campo E, Fisher RI, et al.	N Engl J Med. 2002;346(25):1937-47	2574	135.47
Diffuse large B-cell lymphoma outcome prediction by gene-expression profiling and supervised machine learning	Shipp MA, Ross KN, Tamayo P, Weng AP, Kutok JL, Aguiar RCT, et al.	Nat Med. 2002;8(1):68-74	1647	86.68
CHOP-like chemotherapy plus rituximab versus CHOP-like chemotherapy alone in young patients with good-prognosis diffuse large-B-cell lymphoma: a randomised controlled trial by the MabThera International Trial (MInT) Group	Pfreundschuh M, Trumper L, Osterborg A, Pettengell R, Trneny M, Imrie K, et al.	Lancet Oncol. 2006;7(5):379-91	1380	92.00
Axicabtagene Ciloleucel CAR T-Cell Therapy in Refractory Large B-Cell Lymphoma	Neelapu SS, Locke FL, Bartlett NL, Lekakis LJ, Miklos DB, Jacobson CA, et al.	N Engl J Med. 2017;377(26):2531-44	1228	307.00
EZH2 inhibition as a therapeutic strategy for lymphoma with EZH2-activating mutations.	McCabe MT, Ott HM, Ganji G, Korenchuk S, Thompson C, Van Aller GS, et al.	Nature. 2012;492(7427):108-+	1099	122.11
Stromal Gene Signatures in Large-B-Cell Lymphomas.	Lenz G, Wright G, Dave SS, Xiao W, Powell J, Zhao H, et al.	N Engl J Med. 2008;359(22):2313-23.	1070	82.31
Long-term results of the R-CHOP study in the treatment of elderly patients with diffuse large B-cell lymphoma: A study by the groupe d'Etude des lymphomes de l'adulte	Feugier P, Van Hoof A, Sebban C, Solal-Celigny P, Bouabdallah R, Ferme C, et al.	J Clin Oncol. 2005;23(18):4117-26	991	61.94
Rituximab-CHOP versus CHOP alone or with maintenance rituximab in older patients with diffuse large B-cell lymphoma	Habermann TM, Weller EA, Morrison VA, Gascoyne RD, Cassileth PA, Cohn JB, et al.	J Clin Oncol. 2006;24(19):3121-7	959	63.93

**Table 5:** Citation situation from 1999 to 2021

Items	Global
total	7255
Total cited times	169930
Removes the number of cited times of self-citing	127730
Application documents	70841
The number of citation times cited is removed	65361
Average number of citations per item	23.33
h-index	154

**Table 6:** Ranking of countries in terms of the number of publications of different types of treatments for DLBCL

Ranking of treatment modalities	Chemotherapy drugs	Small molecule compound	Monoclonal antibodies	Cell therapy	Transplant
First	USA	USA	USA	USA	USA
Second	China	China	France	China	UK
Third	Italy	Spain	UK	Japan	Italy
Fourth	France	Germany	Japan	Italy	France
Fifth	Germany	Canada	Germany	France	Germany
Sixth	Japan	Poland	Italy	Germany	Spain
Seventh	UK	Japan	China	UK	Canada
Eighth	Canada	Belgium	Spain	Canada	Australia
Ninth	Spain	Italy	Switzerland	Spain	Japan
Tenth	Switzerland	Switzerland	Canada	Switzerland	China
